# Von-Hipple Lindau syndrome with family history: a case report and seventeen years follow-up study

**DOI:** 10.3389/fonc.2024.1360942

**Published:** 2024-03-26

**Authors:** XueMei Fan, Shuai Wang, Tianwen Chen, Wei Hu, Hui Yang

**Affiliations:** ^1^ Department of Neurology, Affiliated Hangzhou First People’s Hospital, Westlake University School of Medicine, Hangzhou, China; ^2^ Department of Critical Care Medicine, Affiliated Hangzhou First People’s Hospital, Westlake University School of Medicine, Hangzhou, China

**Keywords:** 13588427516, 0531-56006952. 2 Von-Hippel Lindau syndrome, VHL gene, gene mutation, exome sequencing, pedigree analysis

## Abstract

**Background:**

Von-Hipple Lindau syndrome is an uncommon autosomal dominant disorder. 17 years ago we diagnosed a young woman with VHL syndrome validated by Sanger sequencing, her family members were genetically tested as well, and 187 healthy people were randomly selected for VHL genetic testing as controls. We analyze the clinical and genetic characteristics of VHL syndrome in a Chinese lineage and with 17-year follow-up.

**Case presentation:**

A woman was finally diagnosed with VHL syndrome due to the detection of a missense mutation c.353T > C in exon 2 of the short arm of chromosome 3, which resulted in a leucine substitution at amino acid 118 of the encoded protein by a proline, which may be thought the main cause of the disease. The same mutation was observed in two other family members, their clinical symptoms are not entirely identical. However, this mutation was not found in other family members or 187 healthy controls. She clinically presented with central nervous system hemangioblastomas, clear renal cell carcinoma, and pancreatic neuroendocrine neoplasms, despite the multi-organ involvement and several relapses during the disease, the patients survive well for she was treated with aggressive surgery early in the course of the plaguing symptoms, whereas patients who are not aggressively treated have a poorer prognosis.

**Conclusion:**

The clinical presentation of VHL syndrome is atypical, and early identification and treatment of VHL syndrome is possible by genetic testing techniques. Multiple relapses occurred during the course of the disease, but early diagnosis and aggressive treatment allowed the patients to survive well.

## 1 Introduction

Von-Hipple Lindau (VHL) syndrome is an uncommon autosomal dominant disorder that results in the manifestation of a multi-organ tumor syndrome. The VHL gene, which is located on chromosome 3, p25-p26, is responsible for this syndrome, and its mutations and deletions can cause abnormal protein expression that leads to tumor invasion and metastasis. The syndrome is characterized by various tumors, including retinal and central nervous system (CNS) hemangioblastomas(HBs), pheochromocytomas, clear renal cell carcinoma (RCC), pancreatic cystic adenoma, endolymphatic sac tumors, epididymal cystadenomas, and cystadenomas of the broad ligament of the uterus (1). The median survival age of VHL syndrome patients is between 42-50 years, with a median survival of 49.4 years for males and 48.4 years for females, and the leading cause of death is usually RCC and CNS tumors and their related complications (2). The majority of VHL syndrome patients show a positive family history of the disease, while *de novo* mutations are rare. In this report, we present the genomic information on VHL in a family whose proband was a 46-year-old woman who was diagnosed with VHL syndrome 17 years ago due to CNS HBs, RCCs, and pancreatic neuroendocrine neoplasms (PanNENs) caused by a VHL gene mutation (c.353T > C). During her genealogical screening, her mother and son were also found to have the causative gene. As a control, we also sequenced the VHL gene in 187 healthy individuals. We then followed and documented this family line for 17 years.

## 2 Case presentation

**Figure 1:**
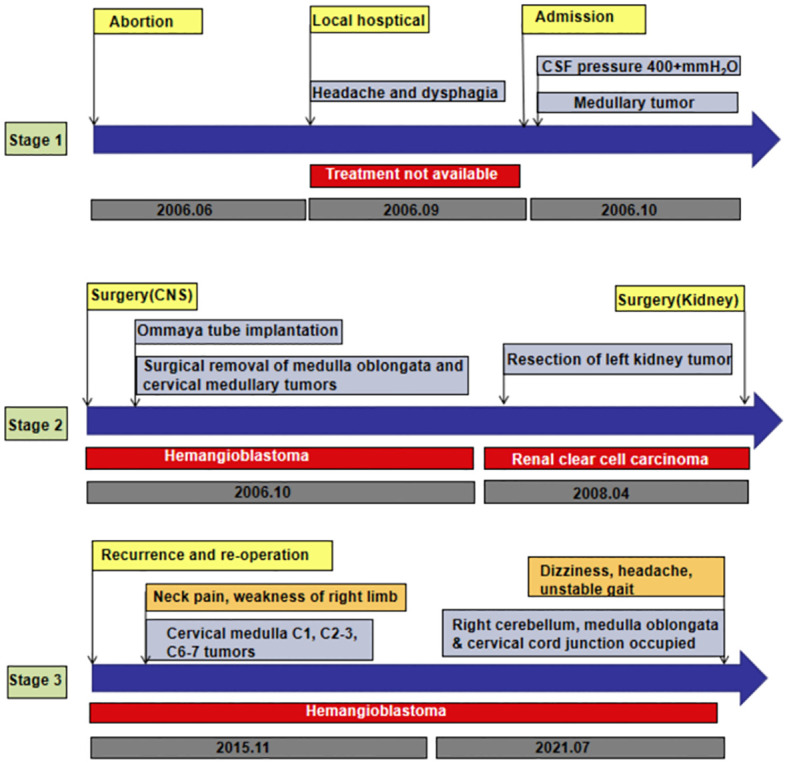
A summary and comparison of CSF characteristics at different stages of the disease.

**Figure 2:**
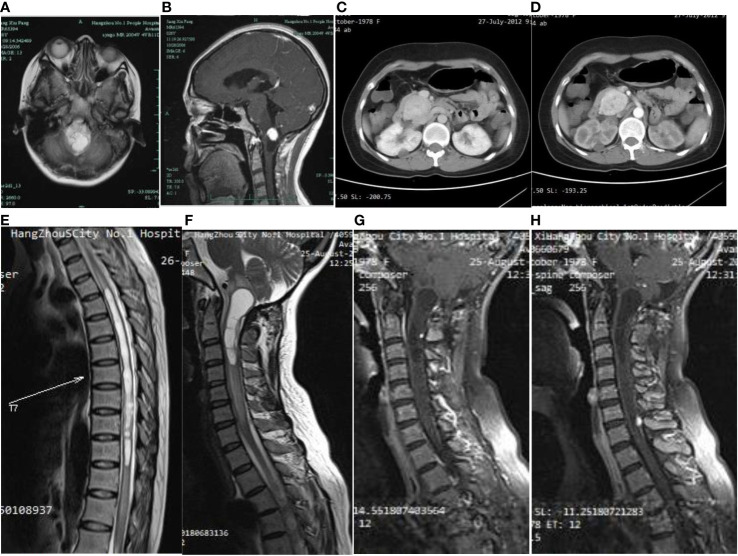
On first admission to the hospital, the brain MRI cystic-solid occupying lesion in the medulla oblongata **(A)**, the solid part showed significant enhancement, while the cystic part was not enhanced **(B)**. Abdominal CT showed an elliptical soft tissue density shadow over the head of the pancreas **(C)**, approximately 4.6×3.6 cm in size, with significant enhancement and several small cyst-like non-enhancing hypodensities in the pancreas **(D)**. A long T2 edema band is seen around the thoracic medulla, which ranges from T4 to T12 segments. Long T1 **(E)** and long T2 **(F)** signal in the medulla oblongata and upper cervical medulla stripes, and foci of punctiform seen after enhancement **(G)**. A round dotted lesion of the posterior spinal cord of the seventh cervical medulla with visible enhancement and localized spinal tail sign **(H)**.

**Figure 3:**
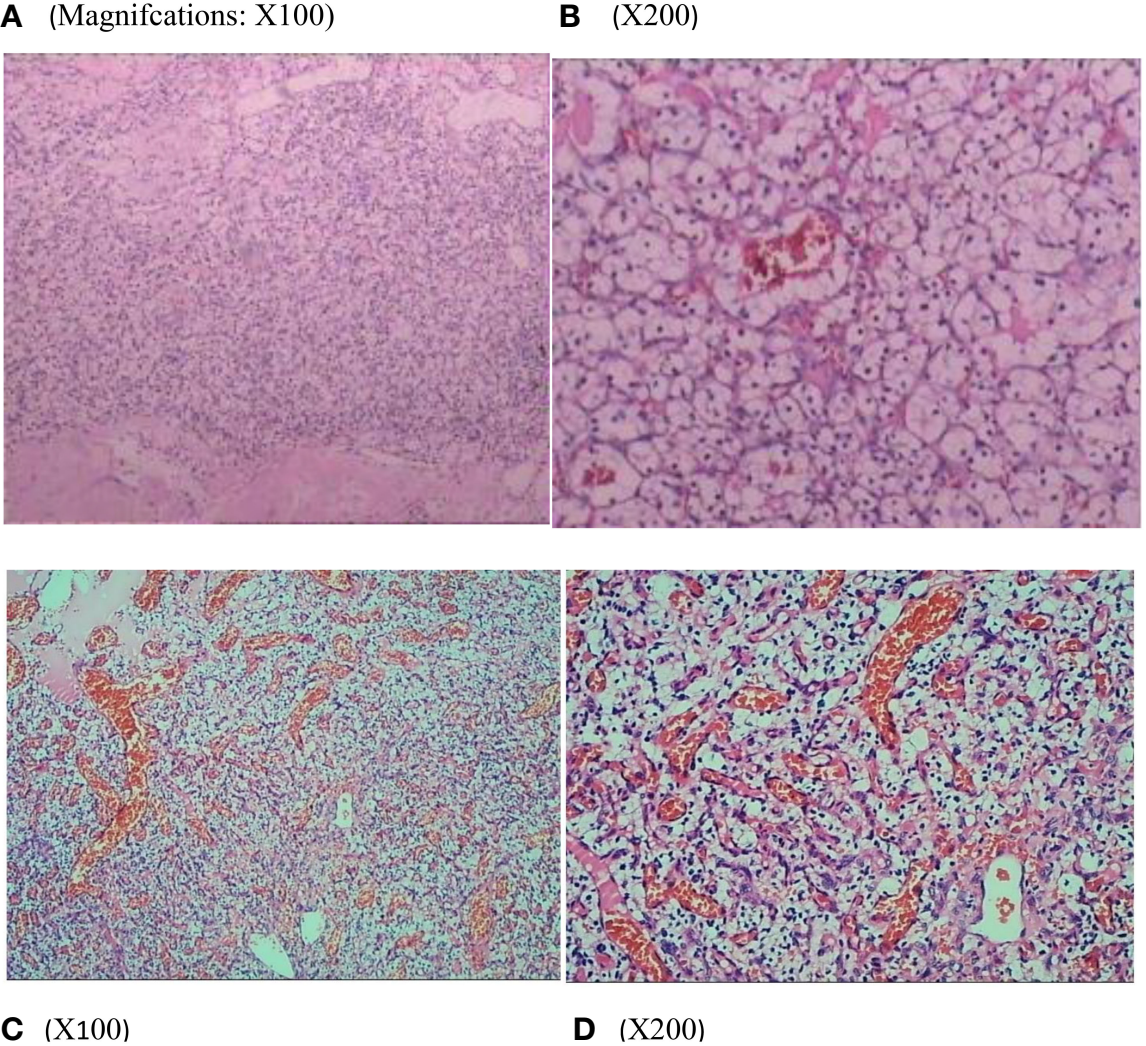
Renal histopathology of the patients showed that the tumor cells were polygonal and columnar in shape, with abundant and transparent cytoplasm, rounded nuclei, partially irregular, uniformly distributed chromatin in the form of fine granules, inconspicuous nucleoli, and localized vascular dilatation in the tumor tissue **(A, B)**. Thoracic medullary mass from the patient’s mother **(C, D)** after surgical resection are subjected to hematoxylin and eosin staining and pathological diagnoses are hemangioblastoma.

A 29-year-old woman underwent progressive worsening headaches and difficulty swallowing, then she was admitted to a local hospital. Her laryngoscopy was normal and the gastroscope showed chronic superficial gastritis. Despite receiving symptomatic treatment and being diagnosed with “vascular headache” based on two normal brain CT scans, the patient’s condition did not improve. Two weeks later, the patient was admitted to our hospital’s neurology department, and her clinical course is detailed in **Figure 1**. A neurological examination revealed a positive kernig sign, stiff neck, absent pharyngeal reflex, poor soft palate elevation, and water swallow test level III. The patient has a 6-year-old son and was pregnant again 1 year ago but induced abortion due to fetal death at 16 weeks of gestation. The cerebrospinal fluid (CSF) was colorless and transparent, with a pressure of 400+ mmH2O. CSF analysis revealed the following: karyocytes, 2/μL; red cells, 0/μL; protein, 0.329 g/L (normal range, 0.150-0.450 g/L); glucose, 4.8 mmol/L (normal range, 2.5-4.5 mmol/L); and chloride, 114 mmol/L (normal range, 120-132 mmol/L). No abnormalities were detected in the blood samples aside from high cholesterol levels. A cranial magnetic resonance imaging (MRI) showed space-occupying lesions in the medulla oblongata (**Figures 2A, B**), ultrasound scans revealed multicentric renal cysts in both kidneys, and a cystic solid mixed density mass in the pancreas was later identified as PanNENs through enhanced CT (**Figures 2C, D**), chest radiograph shows thickened pleura, and Sanger sequencing identified a heterozygous mutation site in the VHL gene, leading to a diagnosis of VHL syndrome. The patient underwent Ommaya implantation for the first time in Huashan Hospital Affiliated with Fudan University in 2006, then underwent craniotomy for tumor resection 2 weeks later. 2 masses were resected in the dorsal medulla oblongata and C1 segment of the cervical cord, measured 3×3×1cm, 2×2×1cm, respectively. The histopathological results of the tumor: purplish-brown tissue as seen by the naked eye. Microscopically, foam cells were seen dispersed between CD34, SMA immunolabelled positive vascular tufts, scattered KP1 and LCA positive cells with GFAP positive gliosis at the margins, the pathological diagnosis was hemangioblastoma. She was diagnosed with renal occupancy in 2008 and underwent resection of the target lesion, which was confirmed as RCC by postoperative pathology (**Figures 3A, B**). In 2015, she developed neck pain, weakness of the right limb, and urinary and fecal disorders, then multiple occupying lesions were detected by spinal cord MRI (**Figure 2E**), and a total of 3 masses in C1, C2-3, and C6-7 were surgically resected, measured 2×1×2mm, 3×2×4mm, 2×1.5×1.0mm, respectively. Pathological findings were considered a HBs, homologous with medulla oblongata occupancy. In 2021, the patient suffered dizziness, headache, and unsteady gait again, and imaging revealed space-occupying lesions in the right cerebellar hemisphere, junction of the medulla oblongata and cervical medulla (**Figures 2F–H**), 3 masses were surgically resected, measured 2.5×2×2cm, 1.5×1×1.5cm, 5×6×2mm, which were confirmed to be HBs by postoperative pathology. Up to now, the patient survived without significant discomfort and possessed a complete social function.

### 2.1 Family profile and VHL gene testing

The patient’s maternal grandparents are related as cousins (I 1 and I 2). Her maternal grandfather died at the age of 40 due to severe headaches. In 1987, her mother(II 2) experienced numbness in her left hand and unstable walking, but she did not seek medical attention. Later, she suffered from blindness in her left eye. In 2020, the patient’s mother was diagnosed with a “hemangioma” in the thoracic medulla by post-surgical pathology (**Figures 3C, D**), which occupied the T8-T10 region, resulting in bilateral lower limb paraplegia and urinary and fecal disorders. The patient’s maternal aunt (II 3) was diagnosed with both “renal cyst” and “pancreatic cyst” and underwent a nephrectomy on the left side in her forties. After obtaining consent from the patient and other family members, peripheral blood samples were collected from a total of eight individuals, including the patient, her mother, her son, her brothers and sister, and her three nephews, for VHL gene testing, the family lineage chart is available in **Supplementary Material 1**. Additionally, 187 individuals from the healthy population were selected as normal controls for VHL gene testing. The polymerase chain reaction was employed to amplify the VHL gene exons from genomic DNA, with primer pairs listed in **Table 1**.

**Table 1:** Primer sequences of exons of VHL gene.

Exons	Primer direction	Primer sequences
Exon1	Forward	5′-TGGTCTGGATCGCGGAGGGAAT-3′
	Reverse	5′-GACCGTGCTATCGTCCCTGC-3′
Exon2	Forward	5′-GTGGCTCTTTAACAACCTTTGC-3′
	Reverse	5′-CCTGTACTTACCACAACAACCTTATC-3′
Exon3	Forward	5′-TTCCTTGTACTGAGACCCTAGT-3′
	Reverse	5′-AGCTGAGATGAAAGAGTGTAAGT-3′

### 2.2 Results

**Figure 4:**
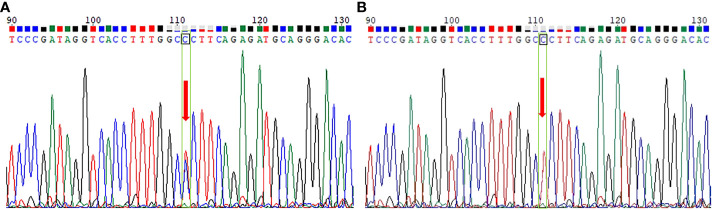
Whole-exome sequencing peak diagram. **(A)** The proband, her mother and son carried a heterozygous variant at the VHL c.353T > C site. **(B)** The other family members and the 187 healthy individuals did not carry this heterozygous mutation.

To confirm the presence of any mutations in the VHL gene, Sanger DNA sequencing was utilized, with each exon analyzed using forward and reverse analysis. Our findings revealed a missense mutation c.353T > C on exon 2 of the short arm in chromosome 3 of the patient. This specific mutation leads to the replacement of leucine with proline at amino acid 118 of the encoded protein, which may primarily account for the VHL syndrome that occurred in the proband (**Figure 4**). Our results indicated that this mutation was also present in the patient’s mother and son. However, this mutation was not detected in other family members and the 187 healthy controls.

### 2.3 Follow up

17 years follow-up was conducted in the family. Despite the multi-organ involvement and several relapses during the disease, early and aggressive surgery led to the patient’s survival well, with no impairment of social behavior. The patient’s mother remained disabled as previously and no new lesions in her brain and spinal cord were detected on her recent physical examination. Her son, who is now 23 years old, remains apparently healthy, he had not undergone whole-body multiorgan imaging until now. The patient’s maternal aunt, a patient with suspected VHL syndrome, was diagnosed with “renal cyst” and “pancreatic cyst”, then underwent a nephrectomy on the left side in her forties. Unfortunately, she did not consent to the invitation to undergo genetic testing and refused to provide detailed physical examination data during our long-term clinical follow-up. Up to now we learned that she did not undergo another surgical procedure and later and died of acute renal failure in 2015 at age 58. The other members of the family remained healthy without similar abnormalities.

## 3 Discussion and conclusions

VHL syndrome is a familial tumor syndrome that progresses systematically and is inherited as an autosomal dominant trait with an incidence of about 1/36,000. This syndrome comprises vascular tumors and similar CNS and organ phenotypes, the most prevalent being retinal and CNS HBs, RCCs, pancreatic islet tumors (1). The pathology is primarily caused by inactivation of the VHL gene, which is located on chromosome 3p25-p26 and encodes a ubiquitin ligase that degrades hypoxia-inducible transcription factor-2alpha (HIF-2α). Inactivation of VHL results in the accumulation of HIF-2α and activation of genes such as vascular endothelial growth factor (VEGF), leading to oncogenesis (3). Over 500 mutations in VHL have been reported worldwide, with varying mutation spectrums depending on ethnicity, including missense mutations, nonsense mutations, small fragment deletions and insertions, large fragment true and splice mutations, etc. Chinese patients commonly exhibit missense mutations in hotspot regions of exons 1 and 3. Two clinical types and multiple subtypes of VHL syndrome can be distinguished (4), type 1: no pheochromocytoma (or less than 10% risk), type 2: with pheochromocytoma (40%–60% risk) type 2A: without renal cancer (rare), type 2B: with renal cancer (common), type 2C: presenting with pheochromocytoma only and without other manifestations of VHL syndrome. A missense mutation in exon 2 of chromosome 3’s short arm at c.353T > C was detected in the patient, she had definite HBs and RCCs, though no pheochromocytoma was found until now, we speculate that she is more likely to be subtype 2B.

CNS HBs are a prevalent tumor in patients with VHL syndrome, found in 60-80% of cases, these tumors are generally benign. According to gene sequencing, exon and intron mutations can both lead to the development of CNS HBs (5). Patients with VHL subtypes 1, 2A, and 2B have a higher risk of CNS disease development or progression (6), this is consistent with our report. Symptoms depend on the anatomical region affected, with mass effects and associated symptoms typically arising from the cystic component (7). The optimal management of CNS HBs is unclear. Surgical resection is the primary treatment; however, the recurrence of the tumor is a major challenge for patients with VHL. Re-surgical treatment may be considered for significant neurological deficit symptoms, hemorrhage of HBs tumor, or new cyst enlargement. For sub-completely resected, unresectable lesions, or cornified HBs diffusion, radiation therapy and/or stereotactic or radiosurgery are valid options, with 5-year tumor control rates of 80-90% (8).

In VHL syndrome, pancreatic lesions are present in approximately 70% of cases (9), consisting of PanNENs and pancreas cystic lesions. Among these, pancreatic cysts are observed in about 50% of VHL patients. Since pancreatic cysts are rarely seen in the general population, their identification during screening examinations can help to identify gene carriers (10, 11). CT examination and enhancement showed multiple cysts in the pancreas, with enhancement in the pancreatic head, which is considered PanNENs, as described in our report. Although PanNENs rarely cause morbidity and mortality, they can transform into malignancy or metastasize, resulting in a poor prognosis. Patients with primary lesions larger than 3 cm, a mutation in exon 3, and a quick tumor doubling time of less than 500 days are at high risk for metastatic disease and should be considered for surgery (12).

Patients in 60% of cases with VHL syndrome present with renal lesions that manifest as renal cysts coexisting with renal neoplasms with renal cysts, as described in our report. The median age at onset of RCC is 37 years (13), and the incidence increases with age, with up to 70% of patients with VHL developing renal cell carcinoma by age 60 and being one of the leading causes of death (14). Notably, renal cell carcinoma may produce multiple hormone-like or cytokine-like bioactive products during all phases of the VHL syndrome course, which leads to a paraneoplastic syndrome (15). Surgical treatment may be considered for renal masses larger than 3 cm in diameter. In individuals with definite VHL syndrome families, an abdominal CT or MRI is recommended every 2 years.

Consanguineous marriages have a high likelihood of passing on the same genetic variant due to shared ancestry. In this case, the patient’s maternal grandfather’s genome information was unavailable because he had passed away, but the headache he experienced raises the possibility of him being the actual patient (I 1) or that the condition existed in a previous generation. Surgical treatment is the primary method of treating VHL syndrome, but advances in precision medicine and sequencing technologies have provided new options for treating VHL syndrome-associated tumors. For instance, belzutifan, an oral small molecule inhibitor of HIF-2α, was approved for treating patients with VHL-related RCC, CNS HBs, or PanNENs who temporarily do not require surgery, marking a milestone in VHL disease-associated tumor treatment (16). In addition, tyrosine kinase inhibitors (TKI), such as sunitinib and pazopanib, which target VEGF receptors, have shown effectiveness in treating VHL-associated RCC, CNS HBs, and PanNENs (17, 18). Furthermore, drugs like bevacizumab, which is an anti-VEGF agent, and the newly discovered novel VHL target zinc fingers and homeoboxes 2 (ZHX2) may be beneficial for patients with VHL-associated RCC (19, 20). Due to some reason, the limitation of this case is that the patient was not treated with novel medications throughout the disease, but rather with multiple surgeries to resolve the plaguing symptoms.

VHL disease is inherited in an autosomal dominant manner, pre-determined patients have a 50% chance of passing the mutant allele to their children. Therefore, it is essential to conduct a detailed genealogical survey of all genetically confirmed patients and provide appropriate genetic counseling. Prenatal diagnosis should be performed for patients with fertility needs. This involves collecting fetal chorionic villi at 11-13 weeks of gestation or amniocentesis at 18-22 weeks of gestation. Additionally, Chinese VHL patients’ offspring have been observed to exhibit earlier onset and more severe symptoms than the parental generation, so monitoring of family patients should begin at an appropriately advanced time. Previous study has suggested that screening protocols for VHL syndrome in mutation-positive children with or without clinical manifestations as follows (4). Retinal angioma should be detected starting in infancy or early childhood, and an ophthalmic examination every 12 months is necessary. As for the CNS hemangioblastoma, initiating surveillance in the first year of life in children with genetically diagnosed VHL syndrome is appropriate, CNS MRI examination every 12-36 months is recommended. Blood pressure monitoring, 24-hour urine samples catecholamine metabolites, and MRI of the abdomen were performed every 12 months from the age of 8 years is useful for screening the occurrence of pheochromocytoma. For children with clinical manifestations, ultrasound and/or MRI of the abdomen every 12 months from the age of 16 years is advantageous for surveillance of Renal carcinoma and pancreatic tumors, however, in children without clinical manifestations, surveillance with ultrasound of the abdomen is recommended from the age of 8 years.

In conclusion, we conducted a follow-up study for 17 years with a VHL family and found among others that VHL syndrome involves multiple organs and that different individuals with the same mutation in the family exhibit different clinical phenotypes. Despite the possibility of experiencing multiple relapses, early and aggressive treatment may improve the prognosis and prolong the survival of patients. As an autosomal dominant disorder, the disease burden of VHL syndrome is undoubtedly enormous. There are few preclinical and clinical studies on this topic, and symptomatic treatment is the mainstay, we expect that in the near future, drugs or treatments targeting genetic targets will suppress the expression of mutated genes before the birth of high-risk infants.

## Data availability statement

Publicly available datasets were analyzed in this study. This data can be found here: [https://www.ncbi.nlm.nih.gov/genbank/BankIt2793623 1 PP262635, BankIt2793623 2 PP262636, BankIt2793623 3 PP262637, BankIt2793623 4 PP262638, BankIt2793623 5 PP262639, BankIt2793623 6 PP262640, BankIt2793623 7 PP262641, BankIt2793623 8 PP262642].

## Ethics statement

The studies involving humans were approved by ethics committee of the First People’s Hospital of Hangzhou. The studies were conducted in accordance with the local legislation and institutional requirements. Written informed consent for participation in this study was provided by the participants’ legal guardians/next of kin. Written informed consent was obtained from the minor(s)’ legal guardian/next of kin for the publication of any potentially identifiable images or data included in this article.

## Author contributions

XF: Writing – original draft. SW: Writing – original draft. TC: Writing – original draft. WH: Writing – review & editing. HY: Writing – review & editing.
