# Supplementary figures and images for: Von-Hipple Lindau syndrome with family history: a case report and seventeen years follow-up study
Source: Front Oncol. 2024 Mar 26;14:1360942. doi: 10.3389/fonc.2024.1360942 (PMC11002081; doi:10.3389/fonc.2024.1360942)

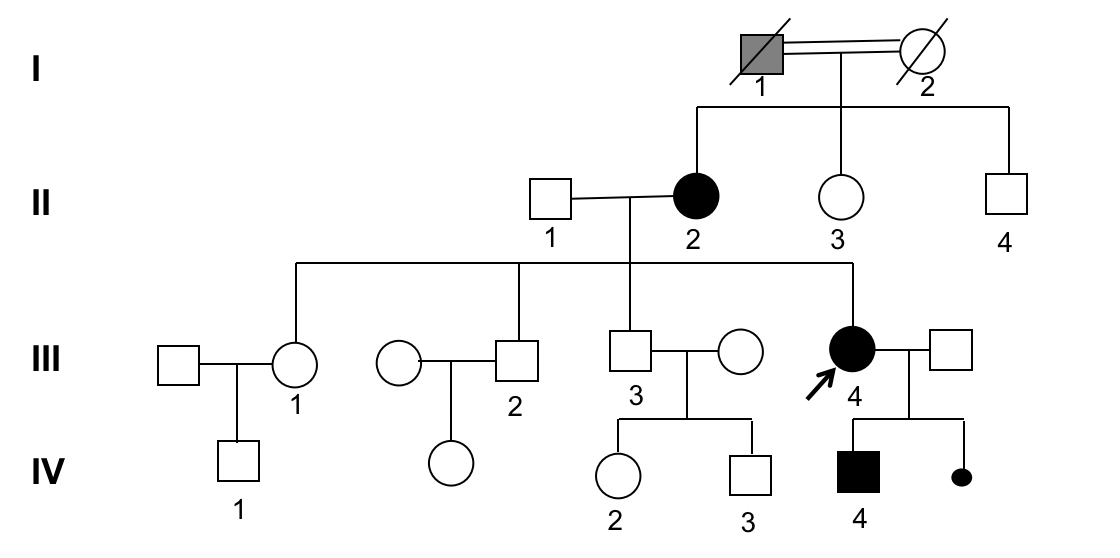

Supplement: Supplementary file 1 [file Image_1.png]
